# Splicing Factor 3B Subunit 1 Interacts with HIV Tat and Plays a Role in Viral Transcription and Reactivation from Latency

**DOI:** 10.1128/mBio.01423-18

**Published:** 2018-11-06

**Authors:** George B. Kyei, Shanshan Meng, Rashmi Ramani, Austin Niu, Chandraiah Lagisetti, Thomas R. Webb, Lee Ratner

**Affiliations:** a Department of Medicine, Washington University School of Medicine, St. Louis, Missouri, USA; b Department of Molecular Microbiology, Washington University School of Medicine, St. Louis, Missouri, USA; c Noguchi Memorial Institute for Medical Research, College of Health Sciences, University of Ghana, Legon, Accra, Ghana; d Division of Biosciences, SRI International, Menlo Park, California, USA; George Mason University; Albert Einstein College of Medicine

**Keywords:** HIV cure, HIV transcription, block and lock, human immunodeficiency virus, latency, reactivation

## Abstract

The main obstacle to an HIV cure is the transcriptionally inert proviruses that persist in resting CD4 T cells and other reservoirs. None of the current approaches has significantly reduced the size of the viral reservoir. Hence, alternative approaches, such as permanent blocking of viral transcription, to achieve a sustained remission, need urgent attention. To identify cellular factors that may be important for this approach, we sought for host targets that when altered could block HIV transcription and reactivation. Here, we identified splicing factor 3B subunit 1 (SF3B1) as a critical HIV dependency factor required for viral replication. SF3B1 is a splicing factor involved in directing chromatin and nascent gene transcripts to appropriate splice sites. Inhibitors of SF3B1 are currently in development for cancer and have been found to be nontoxic to normal cells compared to malignant cells. Knockdown of SF3B1 abrogated HIV replication in all cell types tested. SF3B1 interacted with viral protein Tat *in vitro* and *in vivo*. Genetic or pharmacologic inhibition of SF3B1 prevented Tat-mediated HIV transcription and RNA polymerase II association with the HIV promoter. In addition, an inhibitor of SF3B1 prevented HIV reactivation from latency irrespective of the latency-reversing agent used. The data show that SF3B1 is involved in viral transcription and reactivation from latency and may serve as a therapeutic target in the HIV cure efforts.

## Abstract (precis)

The reason why HIV cannot be cured by current therapy is because of viral persistence in resting T cells. One approach to permanent HIV remission that has received less attention is the so-called “block and lock” approach. The idea behind this approach is that the virus could be permanently disabled in patients if viral genome or surrounding chromatin could be altered to silence the virus, thus enabling patients to stop therapy. In this work, we have identified splicing factor 3B subunit 1 (SF3B1) as a potential target for this approach. SF3B1 interacts with the viral protein Tat, which is critical for viral transcription. Inhibition of SF3B1 prevents HIV transcription and reactivation from latency. Since there are preclinical inhibitors for this protein, our findings could pave the way to silence HIV transcription, potentially leading to prolonged or permanent remission.

## INTRODUCTION

HIV continues to be a global pandemic, accounting for over 1.2 million deaths annually. Treatment with combination antiretroviral therapy (cART) increases life span, recovers CD4 counts, and improves patient well-being. However, cART does not provide a cure for HIV; patients must take medicines for life and deal with side effects and unsustainable costs. In addition, in resource-limited settings where HIV is most prevalent, cART is not accessible to a large number of patients. Hence a cure is urgently needed. The main obstacle to HIV cure is viral latency in resting CD4 T cells and other reservoirs (1–3). Approaches to HIV cure include boosting the host immune system, genetic approaches to disable coreceptors and the viral genome, and using latency-reversing agents (LRAs), the “shock and kill” approach. The shock and kill approach is the most clinically advanced, with several completed preclinical studies (4–7). This approach is based on the idea that latently infected cells can be reactivated to produce virus and in the process die by viral cytopathic effects or immune recognition and clearance. However, so far, this approach has not been able to significantly reduce the size of the viral reservoir, indicating that additional unknown factors involved in viral latency and reactivation need to be identified. To identify novel factors that may be critical for HIV reactivation, we screened a panel of compounds looking for the ability to block HIV reactivation in response to the well-known LRAs. Our rationale was that a target that when modulated can block all known LRAs from reactivating HIV could be a critical host factor required for viral reactivation. Using this approach, we found that an inhibitor of splicing factor 3B subunit 1 (SF3B1), called sudemycin D6, is a potent inhibitor of HIV transcription and reactivation.

SF3B1 is part of the U2 snRNP complex required for mRNA splicing (8–10). Although SF3B1 is traditionally recognized as a splicing factor, recent evidence suggests it may also be involved in chromatin modifications and transcription and may guide nascent transcripts to cotranscriptional splice sites (10–13).

HIV transcription and splicing are a complicated part of the life cycle that has not been successfully targeted for therapy (14, 15). HIV transcription involves the binding of transcription factors like NF-κB and SP-1 to the HIV long terminal repeat (LTR) promoter. Subsequently, the viral protein Tat is crucial for recruitment of P-TEFb (cyclin-dependent kinase 9 [CDK9] and cyclin T1) to the viral mRNA’s stem-bulge-loop structure, TAR, leading off a cascade of events that ensure successful viral transcription (16–18). HIV also depends heavily on alternative splicing to make viral particles (19). At least 40 different spliced mRNA species are produced using four different donor 5′ splice sites and eight 3′ acceptor sites. These multiple mRNA species fall into three main classes: the unspliced (US) 9-kb transcript, which encodes Gag/Gag-Pol; the singly spliced (SS) 4-kb product, which encodes Vif, Vpr, Env/Vpu with a one-exon version of Tat; and the multiply spliced (MS) or completely spliced 1.8-kb product, which encodes Tat, Rev, and Nef (20). Although viral transcription and alternative splicing have not been successfully targeted for clinical use, recent findings show inhibition of these processes could be a viable therapeutic target. First, the small molecule didehydro-cortistatin A binds to Tat and specifically inhibits its transcriptional activities and prevents viral reactivation in latently infected cells (21, 22). Second, IDC16 and its derivatives specifically inhibit the splicing factor SF2/ASF and restrict HIV replication (23). Third, digoxin, a drug that has long been used to treat cardiac arrhythmias, inhibits HIV replication through modulation of viral alternative splicing (24). Molecules that inhibit viral replication through transcriptional and alternative splicing inhibition could also play a crucial role in putting HIV permanently into latency and lead to a functional cure.

Here, we show that pharmacological or genetic inhibition of SF3B1 potently reduces HIV replication. SF3B1 interacts with viral protein Tat and is part of the P-TEFb complex required for HIV transcription. In addition, inhibitors of SF3B1 prevent HIV reactivation in cell line and primary cell models of latency.

## RESULTS

### Depletion of SF3B1 potently inhibits HIV replication.

**FIG 1:**
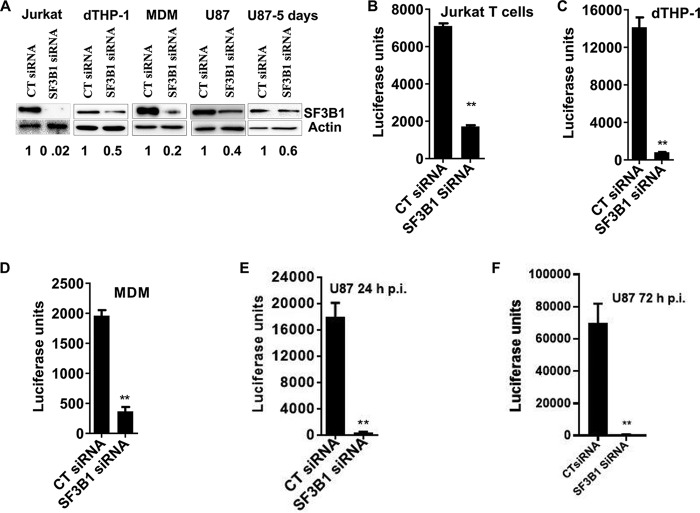
Depletion of SF3B1 inhibits HIV replication in multiple cell types. (A) Western blots showing knockdown of SF3B1 in Jurkat cells, differentiated THP-1 (dTHP-1) cells, monocyte-derived macrophages (MDMs), and U87 cells transfected with control (CT) or SF3B1 siRNA for 48 h (up to 5 days for U87 cells). Levels of knockdown relative to control siRNA are shown below the blots. (B to D) The indicated cells were transfected with control or SF3B1 siRNA. After 48 h, cells were infected with VSV-G-pseudotyped HIV-1 Luc. Twenty-four hours postinfection, cells were lysed for luciferase luminescence and normalized to protein concentration as a measure of HIV replication. (E) U87/CD4/CXCR4 cells were transfected with control or SF3B1 siRNA. After 48 h, cells were infected with replication-competent NL4-3 Luc virus for 24 h, and luciferase measurements were taken as described above. (F) U87/CD4/CXCR4 cells were transfected with control or SF3B1 siRNA. After 48 h, cells were infected with replication-competent NL4-3 Luc virus for 72 h, and luciferase measurements were taken as described above. Data indicate means, and error bars indicate ±standard error of the mean (SEM [*n* = 3]). **, *P* < 0.001, Student’s *t* test. See also Fig. S1.

To evaluate a role for SF3B1 in HIV replication, we used small interfering RNA (siRNA) to knock down the protein in multiple cell types, including Jurkat cells, differentiated THP-1 cells (macrophage-like), and primary monocyte-derived macrophages, cell types extensively used for HIV studies. As shown in Fig. 1A, we achieved variable but significant knockdown of the protein in all cells tested. Cells were infected with HIV-1 *luc* Δ*env* pseudotyped with vesicular stomatitis virus glycoprotein (VSV-G). HIV replication was measured by intracellular luciferase production normalized to the cellular protein concentration. We observed profound reductions in HIV production ranging from 4-fold in Jurkat cells to 17-fold in differentiated THP-1 cells (Fig. 1B to D). The reduction in HIV production was not due to cell death as we performed corresponding protein concentration assays for each experiment and did not see significant reductions in cells treated with SF3B1 siRNA (see Fig. S1A to C in the supplemental material). The level of reduction of HIV replication was more dependent on the cell type than the level of knockdown obtained. To ensure that a fully replication-competent virus that uses the HIV receptor and coreceptor will also be inhibited by SF3B1 knockdown, we used U87/CD4/CXCR4 cells, which are U87 cells that have CD4 and CXCR4 enriched at the surface. Cells with control or SF3B1 siRNA were infected with replication-competent HIV-1 Luc virus, and luciferase measurements of cell lysates were taken after 24 and 72 h to allow for multiple rounds of infection. Again, we observed 40-fold reduction in HIV replication as measured by luciferase, which was maintained up to 72 h postinfection (Fig. 1E and F). Although there was about 20% cell loss in the 72-h infection (5 days post-siRNA transfection), this cannot account for the near complete restriction of HIV replication observed at this time point (Fig. 1F; Fig. S1D and E). Using the Trypan blue exclusion assay, there was no difference in cell viability between control and SF3B1 siRNAs (Fig. S1F).

10.1128/mBio.01423-18.2 FIG S1 (A to E) Protein concentrations after SF3B1 siRNA knockdown. Shown is the total protein concentration for HIV infection assays presented in Fig. 1, measured with the Bradford assay. (F) Cell viability of U87 cells using the Trypan blue exclusion assay. (G and H) Infection assays using HIV-1 Bal virus. TZM-bl cells were transfected with control or SF3B1 siRNA. After 48 h, cells were infected with full-length replication-competent HIV-1 Bal virus for 24 or 48 h. Luciferase readings of cell lysates normalized to total protein concentration were used as a measure of HIV replication. Panel H shows knockdown of SF3B1 at 48 h. (I) Cell viability of TZM-bl cells with the Trypan blue exclusion assay (J) Western blot showing HIV-1 Gag products for the viral infection experiment in Fig. 1C. (K) Knockdown of SF3B1 does not reduce levels of histone H3 or DYRK1A. JLAT10.6 cells were transfected with control or SF3B1 siRNA, and the Western blot was probed for the indicated proteins. (L) Knockdown of SF3B1 reduces levels of heat shock factor 1 (HSF1) in 293T cells. This figure is related to Fig. 1. Download FIG S1, TIF file, 0.3 MB. Copyright © 2018 Kyei et al. 2018 Kyei et al. This content is distributed under the terms of the Creative Commons Attribution 4.0 International license.

Next, we tested the ability of SF3B1 to support the replication of another HIV molecular clone—the R5-tropic HIV-1 Bal. For this, we utilized the TZM-bl cell line, which has HIV LTR-driven luciferase stably transfected and produces luciferase at low levels. Production of Tat by transfection or by successful HIV replication results in increased luciferase production. These cells also have CD4, CCR5, and CXCR4 enriched at the surface. TZM-bl cells transfected with control or SF3B1 siRNA were infected with HIV-1 Bal for up to 48 h, and HIV replication was measured by luciferase luminescence in cell lysates. As shown in Fig. S1G and H, HIV replication was severely restricted in cells transfected with SF3B1 siRNA: up to 20-fold at 48 h postinfection without significant cell death (Fig. S1I). To confirm that the decrease in luciferase was matched by reductions in HIV products, we measured by Western blots the production of HIV Gag in the differentiated THP-1 cells shown in Fig. 1C. As expected, we observed marked reductions in Gag protein production (Fig. S1J). In order to rule out global reduction of cellular proteins upon SF3B1 knockdown, we determined the levels of histone H3 and did not find any difference. In addition, there was no reduction in the levels of the SF3B1 putative kinase DYRK1A (dual-specificity tyrosine phosphorylation-regulated kinase 1A) (9, 10) (Fig. S1K). However, the levels of heat shock factor 1 (HSF1) were reduced with SF3B1 knockdown (Fig. S1L), as previously reported (25). These data show that SF3B1 is required for HIV replication irrespective of the cell type, HIV strain used, or mode of entry of the virus. In addition, depletion of SF3B1 attenuated HIV production without globally reducing cellular protein levels.

### An inhibitor of SF3B1 strongly reduces HIV replication.

**FIG 2:**
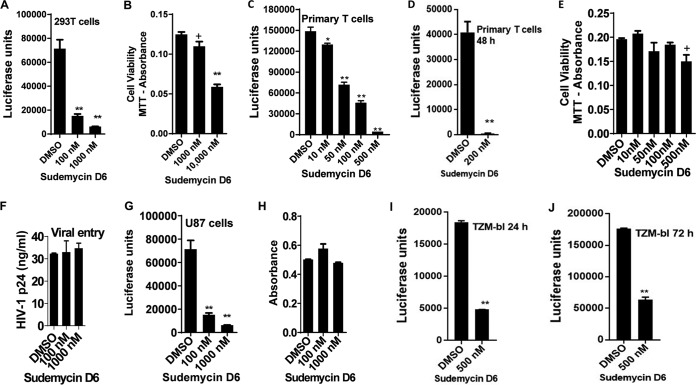
An inhibitor of SF3B1, sudemycin D6, reduces HIV replication. (A) 293T cells were infected with replication-competent VSV-G-pseudotyped HIV-1 Luc with and without sudemycin D6. After 24 h, cells were lysed for luciferase luminescence and normalized to the protein concentration as a measure of HIV replication. (B) 293T cells were treated with increasing concentration of sudemycin D6 for 48 h, and toxicity was determined using the MTT assay (see Materials and Methods). (C and D) Primary CD4^+^ T cells were isolated from donor peripheral blood (one donor for each experiment) and stimulated with PHA/IL-2 for 2 days. Cells were then infected with replication-competent HIV-1 Luc for 24 h (C) or 48 h (D) with and without sudemycin D6, and HIV replication was measured as in panel A above. (E) MTT assay for primary CD4^+^ T cells with increasing concentration of sudemycin D6. (F and G) Full-length HIV was incubated with U87 cells at 4°C for 2 h and washed and incubated at 37°C. Panel F shows the p24 ELISA after 4 h of incubation as a surrogate marker for viral entry. Panel G shows viral replication after 48 h from a concurrent experiment measured by luciferase readings from cell lysates. (H) Measurement of protein concentration in cell lysates for the experiment in panel G. (I and J) TZM-bl cells were infected with HIV-1 Bal virus for 24 h or up to 72 h with either DMSO or sudemycin D6. Cells were lysed, and luciferase measurements were taken to measure HIV replication. Data indicate means, and error bars indicate ±SEM (*n* = 3). **, *P* < 0.001; *, *P* < 0.05; +, *P* > 0.05, ANOVA for multiple comparisons, with Student’s *t* test for pairwise comparisons. See also Fig. S2.

Next, we employed sudemycins, known pharmacological inhibitors of SF3B1, to validate our siRNA knockdown results. Sudemycins are synthetic analogs of natural compounds derived from Pseudomonas and Streptomyces with better stability *in vivo* and excellent bioavailability (26, 27). These compounds specifically bind to SF3B1 to inhibit its activity (11, 28). Therefore, we tested if sudemycin D6, one of the most potent sudemycins, can inhibit HIV replication. First, we infected 293T cells with VSV-G-pseudotyped HIV-1 *luc* Δ*env* in the presence of dimethyl sulfoxide (DMSO) or increasing concentrations of sudemycin D6. As shown in Fig. 2A, treatment with sudemycin reduced HIV production in a dose-dependent manner, 4.7-fold with 100 nM and 11-fold with 1,000 nM, concentrations that were nontoxic to the cells (Fig. 2B). This result was replicated in Jurkat T cells (see Fig. S2A and B in the supplemental material) and differentiated THP-1 cells (Fig. S2C and D). Next, primary T cells from two different donors were treated with sudemycin D6 at a nontoxic concentration and infected with replication-competent HIV-1 Luc for 24 or 48 h to allow for more than a single round of infection. As shown in Fig. 2C and D, there was a profound reduction in HIV replication in a dose-dependent manner, without affecting cell viability, as measured by the MTT [3-(4,5-dimethyl-2-thiazolyl)-2,5-diphenyl-2H-tetrazolium bromide] proliferation assay (Fig. 2E) (29). To ensure that inhibition of SF3B1 did not lead to reduced viral entry, we utilized U87 cells, since the abundance of CD4 and CXCR4 at their surface enhances the entry process. Cells were incubated with replication-competent HIV-1 Luc with and without sudemycin for 2 h at 4°C to enable binding without entry. Cells were then washed vigorously three times and allowed to incubate for 4 h. At 4 h, we determined intracellular p24 and saw no difference, indicating there was no difference in entry (Fig. 2F). However, after 48 h, we noted marked reductions in HIV production (Fig. 2G) without changes in overall protein levels in the cell (Fig. 2H). Finally, we performed a time course infection experiments in TZM-bl cells using HIV-1 Bal virus. Again, we found significant reductions in HIV replication up to 72 h postinfection (Fig. 2I and J; Fig. S2E) without significant cell loss (Fig. S2F). For ongoing multiple rounds of infection, the presence of sudemycin D6 was required to maintain suppression of virus. When the drug was removed, viral production gradually went up over time (Fig. S2G). These results show that, like siRNA depletion, pharmacological inhibition of SF3B1, at doses nontoxic to cells, reduces HIV replication. Taken together, these data show that successful HIV replication is dependent on functional SF3B1.

10.1128/mBio.01423-18.3 FIG S2 (A) Jurkat cells were infected with HIV-1 Luc and incubated with increasing concentrations of sudemycin D6 for 24 h. HIV replication was measured as luciferase luminescence from cell lysates. (B) Total protein concentration in Jurkat cells upon HIV infection with sudemycin D6. (C and D) Differentiated THP-1 cells were infected with HIV-1 Luc for 24 h with increasing concentrations of sudeymcin D6 (C), and a toxicity assay was performed for a similar experiment (D). (E) TZM-bl cells were infected with HIV-1 Bal for up to 72 h with or without sudemycin D6. Luciferase units were normalized to DMSO for easy comparison of the three time points. The same experiment is shown in Fig. 2I and J. (F) Cellular toxicity normalized to DMSO for the experiment in panel E. (G) U87 cells were infected with replication-competent HIV-1 Luc with or without sudemycin D6. Drug was removed after 24 h, and HIV replication was measured with luciferase luminescence in a time course. This figure is related to Fig. 2. Download FIG S2, TIF file, 0.2 MB. Copyright © 2018 Kyei et al. 2018 Kyei et al. This content is distributed under the terms of the Creative Commons Attribution 4.0 International license.

### Depletion or inhibition of SF3B1 blocks Tat-mediated HIV transcription.

**FIG 3:**
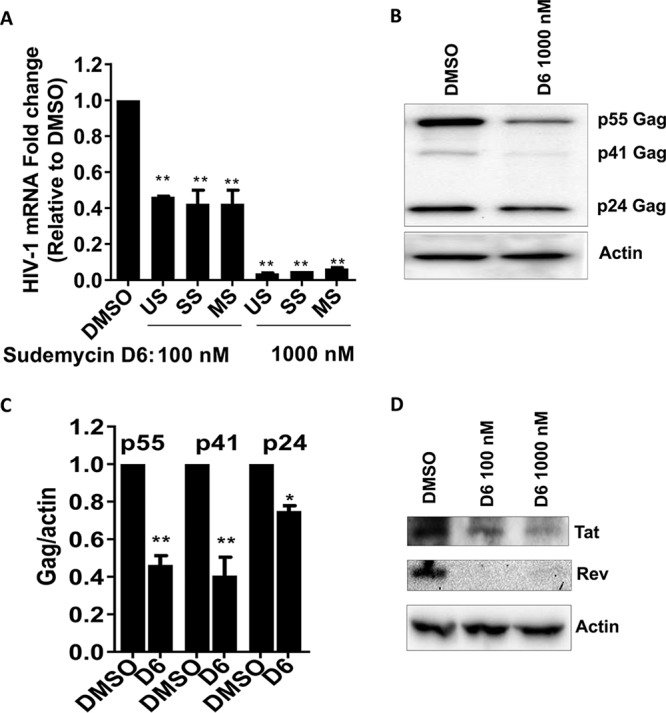
Inhibition of SF3B1 reduces HIV products. (A) U87/CD4/CXCR4 cells were infected with replication-competent HIV-1 for 24 h. Total cellular RNA was obtained, and qRT-PCR was performed for the unspliced (US), singly spliced (SS), and multiply spliced (MS) forms of HIV using specific primers. RNA fold change was calculated relative to DMSO treatment. (B) 293T cells were infected with HIV Δ*env* for 24 h in the presence of DMSO or sudemycin D6. Western blots were performed on cell lysates for HIV Gag products. Three experiments were quantitated (C). (D) 293T cells were infected with HIV Δ*env* for 24 h in the presence of DMSO or sudemycin D6. Western blots were performed on cell lysates for Tat and Rev. Data indicate means, and error bars indicate ± SEM (*n* = 3). **, *P* < 0.001; *, *P* < 0.05, ANOVA for multiple comparisons, with Student’s *t* test for pairwise comparisons.

Since SF3B1 is known to mediate gene splicing, we sought to determine what effect it has on HIV splicing. To do this, we isolated RNA from HIV-infected U87/CXCR4 cells treated with either DMSO or sudemycin D6. Using well-described specific primers for unspliced (US), multiply spliced (MS), and singly spliced (SS) forms of HIV, we quantified the abundance of these splice forms of HIV by quantitative reverse transcription-PCR (qRT-PCR). We expected sudemycin D6 to have differential effect on the various splice forms of HIV, as has been the case for multiple splicing inhibitors (23, 24). Surprisingly, we observed 2- and 14-fold reductions in all three HIV-1 mRNA forms with 100 nM and 1 μM sudemycin D6, respectively (Fig. 3A). To confirm that inhibition of SF3B1 abrogates the production of all HIV splice forms, we used Western blots to probe for HIV proteins produced by the different mRNA products. As shown in Fig. 3B and C, production of HIV-1 Gag (produced from the US transcript) was markedly reduced. In addition, production of Tat (from both the SS and MS transcripts) and Rev (from the MS transcript) was uniformly reduced (Fig. 3D). This result indicates that the effect of SF3B1 on HIV replication may be at the level of transcription.

**FIG 4:**
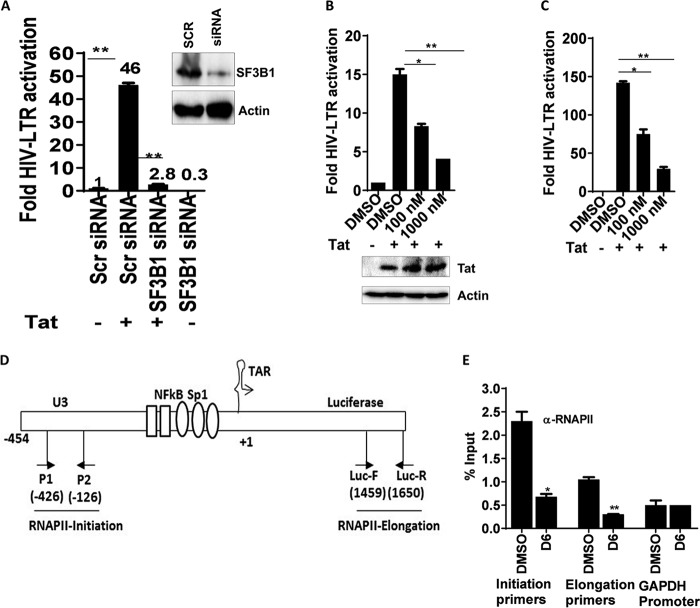
Inhibition of SF3B1 abrogates Tat-mediated HIV transcription. (A) TZM-bl cells were transfected with scramble or SF3B1 siRNA. After 24 h, cells were recounted and transfected with 0.2 μg of full-length HA-Tat plasmid for another 24 h, and luciferase measurements were obtained from the cell lysates. The inset is a Western blot showing knockdown of SF3B1 in TZM-bl cells. (B and C) TZM-bl cells were transfected with 0.1 μg (B) or 1 μg (C) of HA-Tat for 24 h with and without sudemycin D6. Luciferase measurements from the cell lysates normalized to total protein concentration are shown. The Western blot shows levels of HA-Tat expressed in the cell lysates. (D) Primer positions on the HIV LTR used for ChIP experiments. (E) Inhibition of SF3B1 reduces RNAPII association with the HIV LTR. TZM-bl cells were transfected with HA-Tat with or without 1,000 nM sudemycin D6 (D6) for 24 h, and RNAPII ChIP was performed as described in Materials and Methods. RNAPII association with the HIV promoter and luciferase was determined using the primers indicated and expressed as percentage of input. Data indicate means, and error bars indicate ±SEM (*n* = 3). **, *P* < 0.001; *, *P* < 0.05, ANOVA for multiple comparisons, with Student’s *t* test for pairwise comparisons. See also Fig. S3.

To determine whether SF3B1 has a role in HIV-1 transcription, we utilized the TZM-bl cell line. Transfection of these cells with a plasmid that expresses HIV-1 Tat increases luciferase activity in a dose-dependent manner. Knockdown of SF3B1 resulted in marked reduction in Tat-mediated LTR transactivation from 46- to 2.8-fold, a 16-fold reduction in activity (Fig. 4A). Baseline Tat-independent luciferase production was reduced by only 3-fold (Fig. 4A, bars 1 and 4). These data show that Tat-mediated HIV transcription is dependent on a functional SF3B1. Next, we used sudemycin D6 on Tat-transfected TZM-bl cells to assess HIV transcription. Again, inhibition of SF3B1 significantly reduced Tat-mediated transcription of HIV in a dose-dependent manner whether cells were transfected with 0.1 μg or 1 μg of Tat (Fig. 4B and C, respectively). Significantly, treatment of cells with sudemycin D6 did not affect the levels of Tat produced in *trans* from a plasmid (Fig. 4B, Western blot) but did abrogate endogenous Tat production, as observed in Fig. 3D. We then tested the effect of SF3B1 on non-HIV cytomegalovirus (CMV) and NF-κB promoters. In cells stably transfected with either the CMV luciferase (CMV Luc) or NF-κB–luciferase reporter, knockdown of SF3B1 did not result in less transcription. Rather, there were moderate increases in transcription (see Fig. S3A to D in the supplemental material), opposite to what we found with HIV. When another retrovirus human T-cell leukemia virus type 1 (HTLV-1) promoter was similarly evaluated, we found a 2.5-fold decrease (Fig. S3E and F). The effect on HTLV-1 was in the same direction as HIV but much smaller in scale (2.5- versus 16-fold decrease). These data show that SF3B1 controls HIV-1 transcription, has minimal effect on HTLV-1, and does not support nonretrovirus transcription.

10.1128/mBio.01423-18.4 FIG S3 (A and B) Response of CMV promoter to SF3B1 knockdown. HeLa cells stably transfected with the CMV Luc promoter were transfected with control or SF3B1 siRNA for 48 h. Luciferase units in cell lysates normalized to total protein concentration were used as a measure of transcription. Panel B shows knockdown of SF3B1 in these cells. (C and D) Response of the NF-κB promoter to SF3B1 knockdown. HeLa cells stably transfected with the NF-kB-Luc promoter were transfected with control or SF3B1 siRNA for 48 h and stimulated with TNF-α. Luciferase units in cell lysates normalized to total protein concentration were used as a measure of transcription. Panel D shows knockdown of SF3B1 in these cells. (E and F) Response of the HTLV-1 promoter to SF3B1 knockdown. Jurkat cells stably transfected with the HTLV-1 LTR-Luc promoter were cotransfected with control or SF3B1 siRNA and HTLV-1 Tax plasmid for 48 h. Luciferase units in cell lysates normalized to total protein concentration were used as a measure of HTLV-1 transcription. Panel F shows knockdown of SF3B1 in these cells. This figure is related to Fig. 4. Download FIG S3, TIF file, 0.2 MB. Copyright © 2018 Kyei et al. 2018 Kyei et al. This content is distributed under the terms of the Creative Commons Attribution 4.0 International license.

HIV Tat mediates the recruitment of the P-TEFb protein complex, comprising CDK9 and cyclin T1, to the viral promoter, leading to phosphorylation of the RNA polymerase II (RNAPII) C-terminal domain. Subsequently, RNAPII processivity at the HIV promoter increases (30–32). To test whether RNAPII association with the HIV promoter regions is reduced upon SF3B1 inhibition, we used TZM-bl cells for chromatin immunoprecipitation (ChIP) studies to determine the occupancy of RNAPII at the HIV promoter. Cells were transfected with a plasmid expressing Tat and incubated with DMSO or sudemycin, and ChIP was performed with RNAPII antibody followed by qPCR using primers for HIV promoter initiation and elongation (Fig. 4D). Sudemycin D6 reduced the amount of RNAPII associated with HIV-1 promoter and elongation sites by 3-fold. In contrast, RNAPII engagement of the GAPDH (glyceraldehyde-3-phosphate dehydrogenase) promoter was not affected (Fig. 4E). Taken together, our data show that inhibition of SF3B1 severely attenuates Tat-mediated HIV transcription and reduces RNAPII association with the HIV promoter.

### SF3B1 interacts with Tat and the P-TEFb complex.

**FIG 5:**
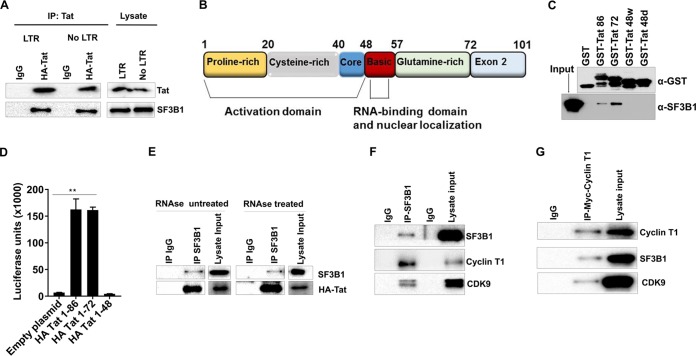
SF3B1 interacts with Tat and the cyclin T1/CDK9 complex independent of RNA. (A) 293T cells were cotransfected with full-length HA-Tat with control plasmid or HA-Tat with HIV-1 LTR plasmid. Immunoprecipitation (IP) was performed with HA antibody, and blots were probed with SF3B1 or Tat antibodies. One of three independent interaction experiments is shown. (B) Domains of HIV-1 Tat, adapted from reference 31. (C) GST-Tat mutants (described in text) were purified from E. coli and incubated with whole-cell lysate. Western blots were probed for endogenous SF3B1. One of four independent experiments is shown. (D) HAT-Tat mutants were transfected into TZM-bl cells for 24 h. Cells were lysed, and luciferase readings were taken as a measure of HIV transcription. (E) TZM-bl cells were transfected with full-length HA-Tat. Cell lysates were treated with or without RNase H. IP of SF3B1 was performed, and blots were probed for Tat. (F) Nuclei were isolated from untransfected JLAT10.6 cells, and IP was performed with SF3B1 antibody. Western blots were probed for the indicated proteins. (G) 293T cells were transfected with Myc-cyclin T1 for 24 h. IP was performed with monoclonal Myc antibody, and the Western blot was probed with cyclin T1, SF3B1, and CDK9 antibodies. Representative blots for two independent experiments are shown for panels E to G. Data indicate means, and error bars indicate ±SEM (*n* = 3). **, *P* < 0.001, ANOVA. See also Fig. S4.

SF3B1 knockdown disproportionately affected Tat-mediated HIV transcription and not endogenous transcription (16-fold versus 3-fold [Fig. 4A]). Therefore, we wondered if SF3B1 interacts with Tat. First, we transfected full-length Tat (HA-Tat, amino acids [aa] 1 to 101) into 293T cells. In cells transfected with either Tat alone or Tat and the HIV promoter (LTR), we could readily pull down endogenous SF3B1 in immunoprecipitates (Fig. 5A). This shows that SF3B1 interacts with Tat independent of the HIV promoter or any HIV products. Next, to confirm the specificity of this interaction, various glutathione *S*-transferase (GST) Tat mutants were employed for *in vitro* interaction studies (Fig. 5B). The following mutants were used: Tat 1–86, which contains all of exon 1 and part of exon 2; Tat 1–72, which contains exon 1 only; Tat 1–48w, which has the wild-type activation domain; and Tat 1–48d, which has a dead activation domain. Tat 1–86 and Tat 1–72 behave like wild-type Tat, while Tat 1–48 lacks the RNA binding domain and the nuclear localization sequence (NLS) (33). We expressed and purified the above GST truncation Tat mutants in Escherichia coli cells, immobilized on beads and incubated with untransfected cell lysates *in vitro*, and probed for SF3B1. As shown in Fig. 5C, there was no binding between GST and SF3B1. However, Tat 1–86 and Tat 1–72 bound SF3B1, while those mutants truncated at position 48 did not. Therefore, even in an *in vitro* setting, SF3B1 interacts with Tat, while Tat mutants that lack the NLS cannot interact. Since SF3B1 is located exclusively in the nucleus, this result confirms the specificity of this interaction. To check the functionality of the Tat mutants, we expressed them in TZM-bl cells. Figure 5D shows that while Tat 88 and Tat 72 performed as expected with 150-fold luciferase production, the mutant that lacks the NLS (and did not bind to SF3B1) could not transactivate transcription. Finally, to ensure that the interaction between SF3B1 is specific and not bridged by small RNAs, we repeated the immunoprecipitation experiments with or without RNase treatment (see Fig. S4 in the supplemental material). As shown in Fig. 5E, the interaction between SF3B1 and Tat was independent of RNA. Thus, our data indicate that SF3B1 control of HIV production may be through its interaction with the critical viral protein Tat.

10.1128/mBio.01423-18.5 FIG S4 RNA degradation in cell lysates prior to the immunoprecipitation experiments in Fig. 5E. HA-Tat-transfected TZM-bl cell lysates were untreated or treated with RNase at 4°C overnight. Afterwards, samples were electrophoresed on 5% Tris-borate-EDTA (TBE) gel. The gel shows degradation of small RNA in the RNase-treated sample. Download FIG S4, TIF file, 0.2 MB. Copyright © 2018 Kyei et al. 2018 Kyei et al. This content is distributed under the terms of the Creative Commons Attribution 4.0 International license.

Since SF3B1 had such a profound effect on Tat-mediated HIV transcription, we tested if SF3B1 could interact with P-TEFb in the absence of Tat. When we pulled down endogenous SF3B1 (in the absence of Tat) in immunoprecipitates, we could readily detect endogenous cyclin T1 and CDK9 (Fig. 5F). To confirm the specificity of these interactions, we expressed Myc-cyclin T1 in 293T cells, immunoprecipitated Myc, and probed for CDK9 and SF3B1. Again, we could detect both CDK9 and SF3B1 (Fig. 5G). These results show that SF3B1 interacts with the P-TEFb complex and that its effect on HIV transcription is likely mediated through these interactions.

### Inhibition of SF3B1 abrogates HIV reactivation from latency.

**FIG 6:**
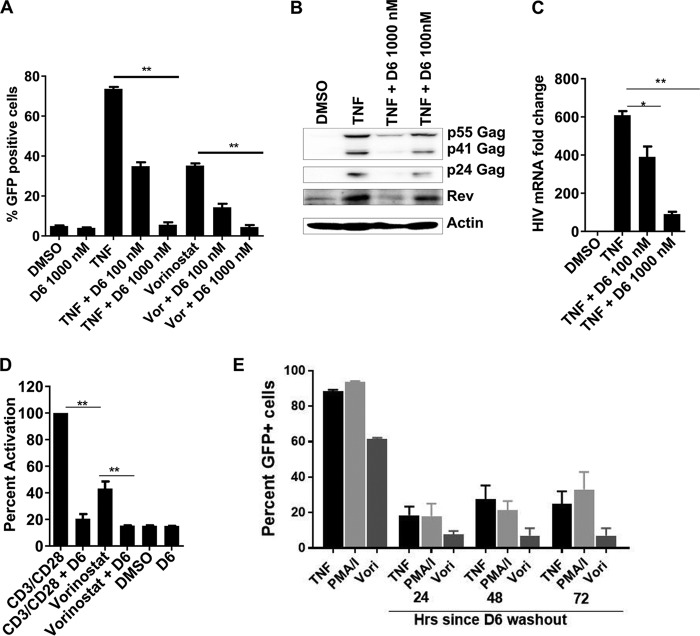
Inhibition of SF3B1 prevents HIV reactivation from latency. (A) JLAT10.6 cells were incubated with the indicated compounds for 24 h, and FACS was performed to detect live GFP-positive cells as a measure of HIV reactivation from latency. (B) JLAT10.6 cells were incubated with the indicated compounds for 24 h. Cells were lysed and resolved on Western blots for the HIV products. A representative blot of 3 independent experiments is shown. (C) qRT-PCR for a similar experiment in panel B. Fold change is relative to DMSO treatment. (D) Inhibition of HIV reactivation in the Greene model of latency. Resting CD4^+^ T cells were infected with HIV-1 Luc, treated with protease inhibitor darunavir for 2 days. Cells were incubated with the compounds shown for 24 h in the presence of raltegravir. (See Materials and Methods for details.) HIV reactivation was measured by luciferase-based luminescence in the cell lysates normalized to protein concentration. (E) JLAT10.6 cells were treated with 5 μM sudemycin D6 for 18 h. Cells were washed and allowed to recover for 24 h. At 24, 48, or 72 h after drug washout, the cells were treated with LRAs for 24 h. HIV reactivation was measured with FACS to detect live cells expressing GFP. Data indicate means, and error bars indicate ±SEM (*n* = 3). **, *P* < 0.001; *, *P* < 0.05, ANOVA.

Because knockdown or inhibition of SF3B1 prevented HIV transcription, we speculated that it may affect HIV reactivation from latency. To test this idea, we used the JLAT10.6 HIV-1 latency model. In this cell line, HIV-1 Δ*env* from the NL4-3 backbone with green fluorescent protein (GFP) replacing the Nef gene, is stably integrated into the genome and is expressed at undetectable levels. Upon HIV reactivation with latency-reversing agents, like tumor necrosis factor alpha (TNF-α) viral replication results in the expression of GFP that can be measured by fluorescence-activated cell sorter (FACS) analysis (34, 35). Treatment of JLAT10.6 cells with 100 nM sudemycin D6 reduced TNF-α-induced HIV-1 reactivation from 74% to 35%. With 1 μM sudemycin D6, HIV-1 induction by TNF-α was completely abolished. Similar results were obtained for the histone deacetylase inhibitor vorinostat (Fig. 6A). To ensure that this reduction in GFP-positive cells correlated with levels of HIV products, we used immunoblots to measure HIV-1 Gag and Rev production. As shown in Fig. 6B, treatment with sudemycin D6 resulted in marked reductions in HIV-1 Gag, and Rev. Finally, we measured HIV-1 mRNA levels by qRT-PCR upon reactivation of HIV in JLAT10.6 cells and observed a 6.7-fold reduction in HIV mRNA with 1 μM sudemycin D6 (Fig. 6C). To confirm our reactivation data, we used the primary cell latency model described by Greene and colleagues (36). Briefly, resting CD4 T cells were isolated by negative selection, infected with an HIV-1 Luc reporter virus by spinoculation for 2 h, washed, and incubated for 72 h with the HIV-1 protease inhibitor darunavir. Viral reactivation with T-cell receptor agonist CD3/CD28 or vorinostat was done with or without sudemycin D6, and intracellular luciferase readings were obtained as a measure of reactivation. With either stimulant, sudemycin D6 prevented HIV-1 reactivation, resulting in levels similar to those seen in cells treated with DMSO (Fig. 6D). Finally, JLAT10.6 cells were treated with high concentration of sudemycin D6 or DMSO for 18 h. Drug was washed off, and stimulation with LRAs was performed at different time points to determine how long the suppression of reactivation will last. For JLAT10.6 cells that have HIV already integrated, there was sustained suppression of HIV reactivation after sudemycin D6 removal (Fig. 6E). These data show that SF3B1 controls HIV replication at a postintegration step, and its inhibition can suppress HIV reactivation, irrespective of the latency-reversing agent used.

## DISCUSSION

In this study, we identified SF3B1 as a critical HIV dependency factor that interacts with Tat and the P-TEFb complex. Knockdown or inhibition of SF3B1 abrogates HIV transcription and reactivation from latency. SF3B1 is an important protein in pre-mRNA splicing. Thus, we were initially surprised to find that most of the effect on HIV seemed to be on transcription. However, recent evidence shows that SF3B1 is intimately involved with chromatin and may be required as a mediator between transcription and splicing (11, 13, 37). In that case, the effect on HIV observed in this study will be expected. Although this study does not rule out an SF3B1 effect on HIV splicing, the evidence that it controls viral transcription is much stronger. First, it interacted with Tat, which is critical for viral transcription. Second, knockdown or inhibition by sudemycin D6 abrogated LTR transcription in the presence of Tat and prevented Tat-mediated RNAPII interaction with the viral promoter. Finally, our finding that the inhibitor of SF3B1 reduced the levels of Tat produced from the HIV promoter, but not from Tat expressed in *trans*, indicates that the interaction between SF3B1 and Tat is specific. Moreover, we did not find reduction in CMV or NF-κB promoter transcription. Although knockdown of SF3B1 reduced HTLV-1 transcription, the effect was modest compared to that in HIV, a finding that could be explained by the fact that HIV Tat and HTLV Tax are controlled by different transcription elements (38).

We found that SF3B1 interacts with Tat *in vivo* and *in vitro*. While more studies are needed to define the exact amino acid motifs required for this interaction, our mutational analysis showed that the Tat amino acids 48 to 72 are important for SF3B1 binding. More importantly, this interaction is not likely to be mediated by TAR or other RNAs, as RNase treatment of lysates could not abrogate the interaction. Whether the interaction between SF3B1 and Tat is direct or through the P-TEFb complex remains to be determined. SF3B1 interacted with Tat and the P-TEFb complex in the absence of the HIV promoter. This suggests that it may be involved in efficient Tat or P-TEFb recruitment to the viral promoter. Ongoing work is looking at the specific role played by SF3B1 in these complex interactions.

Inhibitors of SF3B1 abrogated HIV reactivation from latency in the JLAT10.6 cell model and in a primary cell model of latency, irrespective of the latency-reversing agent used. Suppression of reactivation was maintained for 72 h after drug removal. While this finding could be due to modification of epigenetics to maintain latency, it is could also be the result of sustained binding between sudemycin D6 and SF3B1. Of note, when sudemycin D6 was removed from cells undergoing multiple rounds of infection, there was a steady rise in HIV replication over time. This suggests that SF3B1 is more likely to be involved in maintenance rather than establishment of latency. Long-term experiments using primary T cells from patients suppressed on cART are ongoing to clarify the role of SF3B1 in latency. The fact the LRAs utilize different mechanisms of action suggests that SF3B1 is required for a common pathway required for reactivation. This makes SF3B1 a potential candidate for the “block and lock” approach to HIV remission—the idea that targeting the viral or cellular epigenetics could permanently prevent viral reactivation even when cART is withdrawn (39). The observation that SF3B1 inhibition can reduce acetylated histone H3K36me3 levels (11) and the recent finding that H3K36me3 is enriched at HIV integration sites (40) raise the intriguing possibility that SF3B1 may be a central player in viral reactivation, a possibility that is worthy of further investigation.

An important consideration is whether SF3B1 could be a good therapeutic target since it plays such central role in cellular splicing. Interestingly, this protein is involved in leukemias/lymphomas and breast cancer and has become a therapeutic target (41, 42). What is surprising is that inhibitors of SF3B1 have so far not been overtly toxic in mouse models (43, 44). Inhibition of SF3B1 differentially affects tumor cells rather than normal cells (45). In terms of HIV, if the specific interactions with Tat are worked out, then more specific inhibitors could be developed to target that interaction, which will have much less effect on the cellular functions of the protein.

Since the “shock and kill” approach to HIV cure has encountered multiple obstacles (7, 39, 46), it is important to explore alternative scalable approaches like the “block and lock” approach. Here, we suggest that SF3B1 could be an important candidate for the block and lock approach, given its interactions with Tat and with chromatin.

## MATERIALS AND METHODS

### Primary cells and cell lines.

Peripheral blood mononuclear cells were isolated using density gradient centrifugation through a Ficoll-Hypaque gradient (GE Healthcare). The EasySep CD4^+^ T-cell enrichment kit (Stem Cell Technologies) was used to isolate CD4^+^ T cells. Purity of resting CD4^+^ lymphocytes was verified by flow cytometry and was typically greater than 96%. Isolated cells were maintained in RPMI supplemented with 10% fetal bovine serum (FBS). Where needed, CD4^+^ T cells were treated with phytohemagglutinin (PHA [4 μl/ml]) and interleukin-2 (IL-2 [20 U/ml]) for 48 h prior to HIV infections. For the preparation of macrophages, monocyte differentiation was performed for 7 days using 50 ng/ml of recombinant human macrophage colony-stimulating factor. We prepared the Jurkat/HTLV-Luc stable cell. The HeLa/CMV Luc cell line was obtained from Takashi Murakami, Faculty of Medicine, Saitama Medical University, Japan. The HeLa/NF-κB cell line was from Signosis, Inc. (Santa Clara, CA). JLAT10.6, U87CD4/CCR5, U87CD4/CXCR4, and TZM-bl cells were obtained from the NIH AIDS Reagent Program, Division of AIDS, NIAID. Jurkat, THP-1, and 293T cells were obtained from ATCC. JLAT10.6, THP-1, and Jurkat cells were maintained in RPMI supplemented with fetal bovine serum, l-glutamine, sodium pyruvate, and penicillin-streptomycin. 293T and TZM-bl cells were maintained in Dulbecco’s modified Eagle’s medium (DMEM) supplemented with FBS, while for U87/CD4/CXCR4 cells, 1 μl/ml puromycin and 0.3 mg/ml of G418 were added to the supplemented DMEM. Where indicated, THP-1 cell differentiation was performed with 50 ng/ml of phorbol 12-myristate 13-acetate (PMA) for 48 h.

### Plasmids and siRNA transfections.

The HIV-1 *luc* Δ*env* reporter virus is from a pNL4-3 background, with luciferase replacing the Nef gene, as has been previously described (47). The NL4-3 lucmCherry virus was a gift from Warner Greene (36). This virus has all HIV genes intact, including the Nef gene. HIV-1 Bal virus was from the AIDS Reagent Program, contributed by Bryan R. Cullen (48). SF3B1 ON-TARGETplus SMARTpool siRNA cocktail was obtained from Dharmacon (GE Healthcare; catalog no. L-020061-01-0005). The pcDNA3 Tat hemagglutinin (HA [P 29]) was a gift from Matija Peterlin (Addgene plasmid no. 14654) (49). GST Tat mutants were obtained from NIH AIDS Reagent Program, Division of AIDS, NIAID, NIH, from Andrew Rice. The mutants were GST-Tat 1–86R, two exons, wild-type expressed (catalog no. 2367), GST-Tat 1–72R, one exon, wild-type expressed (catalog no. 2364), GST-Tat 1–48w, truncated after amino acid 48 with wild-type activation domain (catalog no. 2344), and GST-Tat 1–48d, truncated at amino acid 48 with a nonfunctional activation domain expressed (catalog no. 2358) (33, 50) HA-Tat mutants were amplified from the GST constructs above and cloned into pCDNA3. All transfections (except for virus generation) were performed with the Lonza nucleoporation protocol according to the manufacturer’s instructions.

### Reagents and antibodies.

Sudemycin D6 was made by SRI International. PHA (L8754), IL-2 (17908-10KU), TNF-α (T6674), and suberanilohydroxamic acid (SAHA [SML0061]) were obtained from Sigma. Raltegravir (S2005) and darunavir (S1620) were purchased from Selleckchem. The cell line Nucleofector kit V (VCA 1003) was from Lonza. Rabbit monoclonal SF3B1 antibody (ab172634), Mouse monoclonal HA antibody (ab18181), SF3B1 polyclonal antibody (ab172634), Rev antibody (ab85529), Tat antibody (ab43014), and histone H3 antibody (ab1791) were from Abcam. Antibody for DYRK1A was from Abnova (H00001859-M01). Myc monoclonal antibody was obtained from Biolegend (626802), and SF3B1 monoclonal antibody was from LSbio (LS-C179473). Antibodies for GST (2622), CDK9 (2316S), and cyclin T1 (81464S), were from Cell Signaling. CD3/CD28 beads (11161D) were obtained from Invitrogen. Femto Supersignal (PI34096) was from Thermo Fisher. Monoclonal RNAPII antibody (05-623) for chromatin immunoprecipitation was obtained from Millipore. Mouse isotype control IgG (5415S) was from Cell Signaling. Goat polyclonal actin (SC1615) and donkey anti-goat IgG-horseradish peroxidase (HRP [SC-2020]) were from Santa Cruz Biotechnology. Monoclonal p24 Gag antibody was obtained through the NIH AIDS Reagent Program, Division of AIDS, NIAID, NIH: anti-HIV-1 p24 hybridoma (183-H12-5C) from Bruce Chesebro (51).

### Virus generation and infections.

HIV-1 *luc* Δ*env* virus pseudotyped with VSV envelope glycoprotein (VSV-G) was produced from cleared supernatants of cotransfected HEK293T cells using Lipofectamine 3000 reagent according to the manufacturer’s instructions (Life Technologies). Replication-competent NL4-3 Luc (36) and HIV-1 Bal viruses were similarly produced without VSV-G. Viral infections were done with 10 to 50 ng virus as determined by p24 capture enzyme-linked immunosorbent assay (ELISA [ABL, Inc.; no. 5421]). Higher concentrations of virus were used for differentiated THP-1 cells and primary macrophages. After 24 to 72 h of infection, cells were lysed with 0.5% Triton X-100, and luciferase-based luminescence was measured. A Bradford bicinchoninic acid (BCA) assay was performed for each well, and luciferase numbers were normalized to protein concentration.

### Treatment of cells with sudemycin D6 and toxicity assays.

Cells were treated with sudemycin D6 for 24 to 72 h, as indicated, or with an equivalent amount of DMSO. For the reactivation assay in the JLAT10.6 cell line, cells were treated with DMSO, 10 ng/ml of TNF-α, or 1 μM vorinostat (SAHA) with and without increasing concentrations of sudemycin D6 for 24 h. DMSO and TNF-α were used as negative and positive controls, respectively. JLAT10.6 cells were treated for 24 h, and fluorescence-activated cell sorter (FACS) analysis was performed for GFP-positive cells using the Becton Dickinson (BD) FACS. Analysis was done using BD’s CellQuest Pro software. The cells typically diverged into two groups on the scatter plot; viable cells were selected across each treatment for analysis. For all other treatments, sudemycin D6 was added with the virus for the duration of the infection unless otherwise stated. For cell toxicity, an MTT assay was used as previously described (29). Assay reagents were obtained from Sigma (CGD1-1KT) and performed according to the manufacturer’s instructions. Briefly, HIV-infected cells exposed to sudemycin D6 for 24 h were incubated with MTT reagent for 3 to 4 h and lysed in a solubilization buffer. The absorbance of each sample was reported by a microplate reader (Tecan), using the Magellan software program at a wavelength of 570 nm with a reference wavelength of 750 nm. For Trypan blue exclusion, the 0.4% dye was diluted 1:1 with cell suspension and counted with hemocytometer and the Countess II FL automated cell counter. The percentage of live cells (unstained) was plotted as shown in the relevant figures.

### Western blots and immunoprecipitations.

Cells were washed in PBS and lysed with buffer containing 0.2% NP-40 and protease inhibitor cocktail (Roche). Five to 20 μg of protein was loaded and separated on a 12.5% SDS-polyacrylamide gel and transferred to nitrocellulose. The membrane was blocked for 1 h at room temperature in 5% milk in PBS-Tween 20 (0.05%) and incubated with antibody overnight at 4°C. The blot was probed with the appropriate horseradish peroxidase-conjugated secondary antibody for 1 h at room temperature and stained with Femto Supersignal. Actin was probed as loading control. ImageJ software was used to quantify blots. For immunoprecipitations, cells were lysed for 1 h on ice with 0.2% Nonidet P-40 in PBS containing protease and phosphatase inhibitors. This was followed by centrifugation to remove cellular debris. Supernatants were incubated with the immunoprecipitating antibody overnight at 4°C, after which the immunocomplexes were captured with protein G-agarose beads (EMD Biosciences) for 1 h. Beads were washed three times with PBS in 0.1% NP-40 and eluted with SDS-PAGE sample buffer for 5 min at 100°C. Western blots were then performed as described above. Where indicated, RNase treatment of cell lysates prior to immunoprecipitation was performed according to the protocol described by Kang et al. (52). GST-Tat mutants were amplified in E. coli with IPTG (isopropyl-β-d-thiogalactopyranoside [BRL catalog no. 5529]) and purified using glutathione Sepharose 4B beads (Pharmacia). Purified conjugated beads were incubated with 293T whole-cell lysates at 4°C overnight, washed, and resolved by SDS-PAGE. Western blots were performed and probed with SF3B1 or GST monoclonal antibody.

### ChIP.

TMZ-bl cells transfected with HA-tagged Tat were treated with DMSO or sudemycin immediately after transfection and incubated for 24 h. ChIP was performed with a ChIP kit from Abcam (ab500) according to the manufacturer’s instructions. Five micrograms of RNAPII antibody or anti-mouse IgG was used for immunoprecipitation. Total chromatin (input) and the immunoprecipitated chromatin were used as the template, and qPCR was performed using the primers indicated in Text S1 in the supplemental material. Immunoprecipitated DNA or input (diluted 1:20) was used for qPCR with primers positioned in the LTR-luciferase (LTR-Luc) promoter as follows: LTR promoter (positions −426 to −126), luciferase open reading frame (ORF) (positions 1459 to 1650), and GAPDH promoter. Primer sequences are shown in Text S1. Data sets were normalized to input values using threshold cycle (*CT*) numbers of cycles as follows: % input = 2^(^*^CT^* ^of input −^ *^CT^* ^of IP)^ × 100, where “*CT* of IP” represents the threshold cycle number of the immunoprecipitated chromatin. The IgG ChIP background percentages ranged from 0.001 to 0.008% and were subtracted from their respective conditions.

10.1128/mBio.01423-18.1 TEXT S1 Primer sequences used in this study. Download Text S1, TIF file, 0.1 MB. Copyright © 2018 Kyei et al. 2018 Kyei et al. This content is distributed under the terms of the Creative Commons Attribution 4.0 International license.

### RNA isolation and qRT-PCR.

U87/CD4/CXCR4 cells were infected with replication-competent HIV for 24 h with and without sudemycin D6. JLAT10.6 cells were treated with DMSO or TNF-α with or without sudemycin D6. Total cell RNA extraction was done with the RNeasy minikit (Qiagen) according to the manufacturer’s instructions. Real-time qRT-PCR was done with the Bio-Rad One-Step RT-PCR kit using the Bio-Rad CFX Connect system with SYBR green. All reaction mixtures included a negative control in which no reverse transcriptase was added. Gene expression was calculated by the comparative threshold cycle method (2^−ΔΔ^*^CT^*) using GAPDH as a control. Primers for the unspliced (US), singly spliced (SS), and multiply spliced (MS) HIV transcripts are shown in Text S1.

### Statistics.

Student’s *t* test was used for pairwise comparisons, and one-way analysis of variance (ANOVA) was performed for multiple comparisons using the Graphpad Prism software. A *P* value of <0.05 was considered significant.
