# Supplementary figures and images for: Splicing Factor 3B Subunit 1 Interacts with HIV Tat and Plays a Role in Viral Transcription and Reactivation from Latency
Source: mBio. 2018 Nov 6;9(6):e01423-18. doi: 10.1128/mBio.01423-18 (PMC6222122; doi:10.1128/mBio.01423-18)

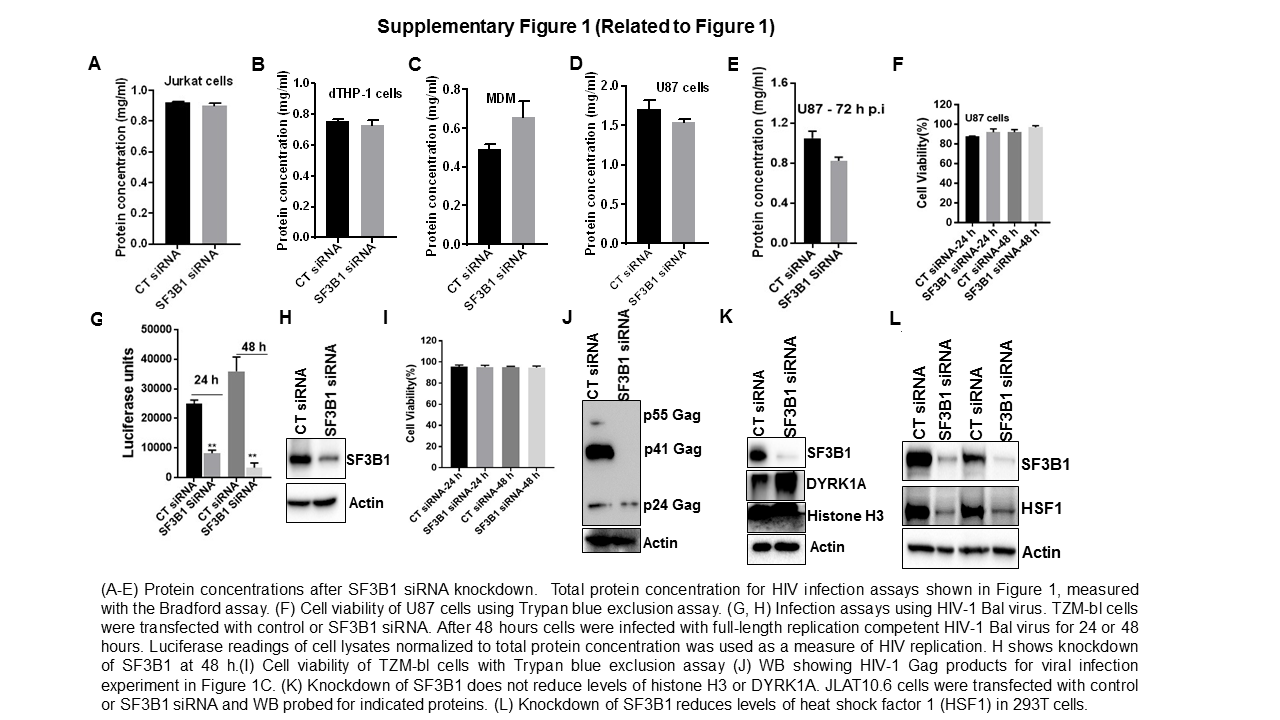

Supplement: FIG S1 [file mbo005184147sf1.tif]

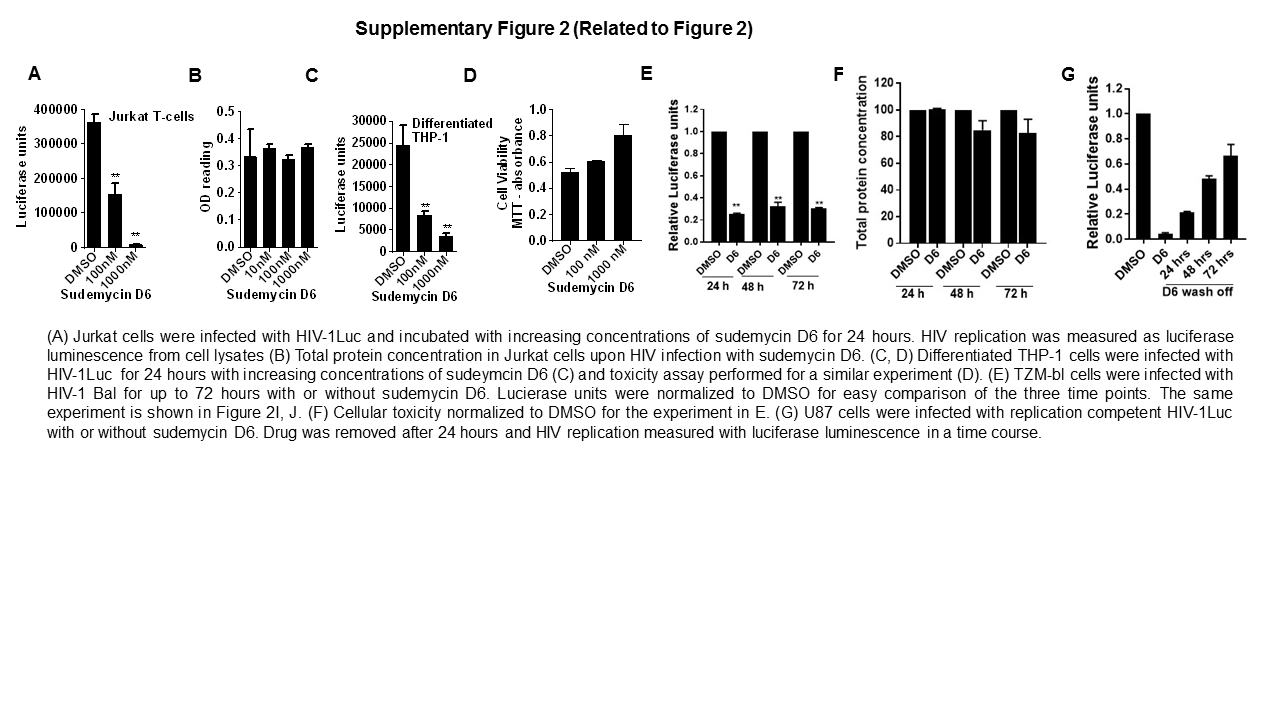

Supplement: FIG S2 [file mbo005184147sf2.tif]

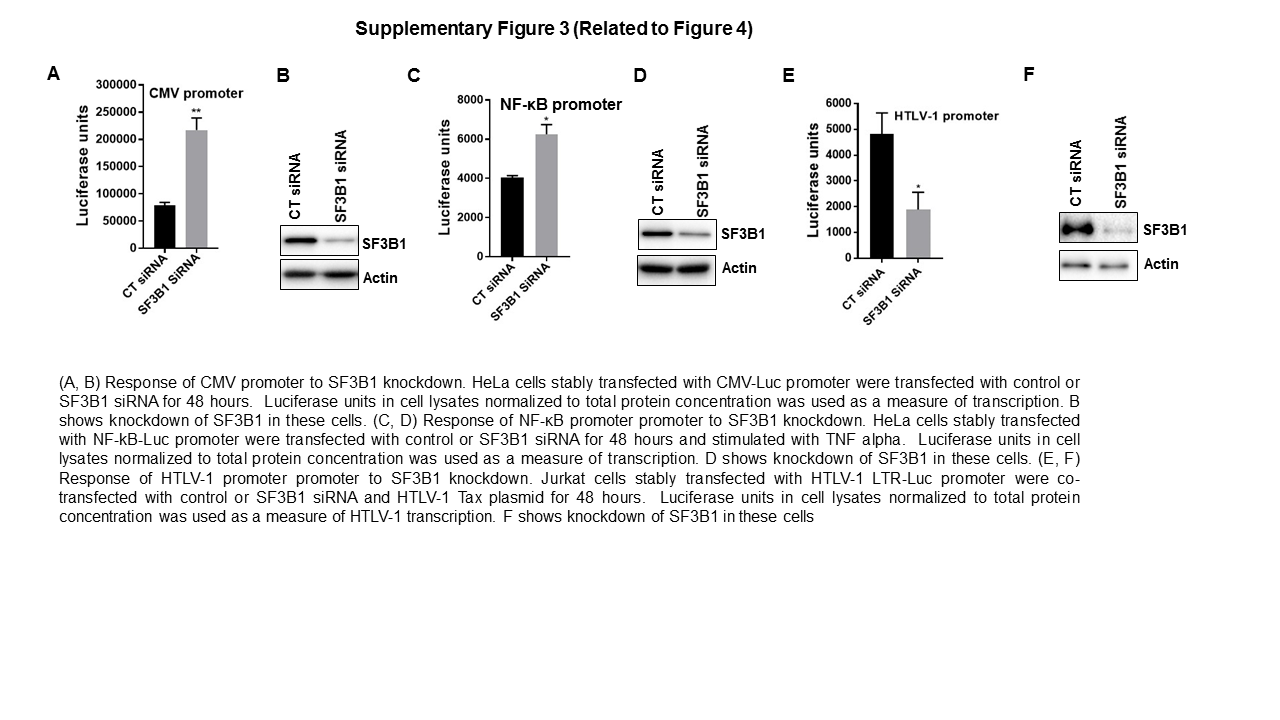

Supplement: FIG S3 [file mbo005184147sf3.tif]

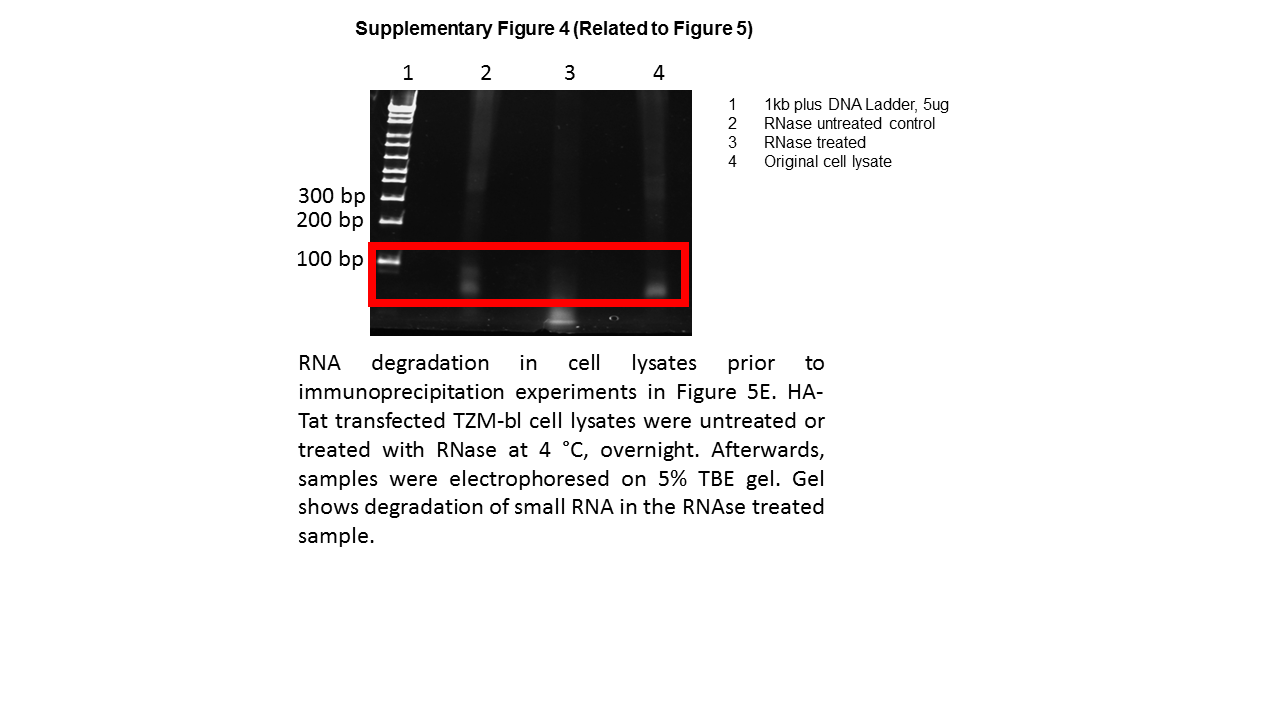

Supplement: FIG S4 [file mbo005184147sf4.tif]

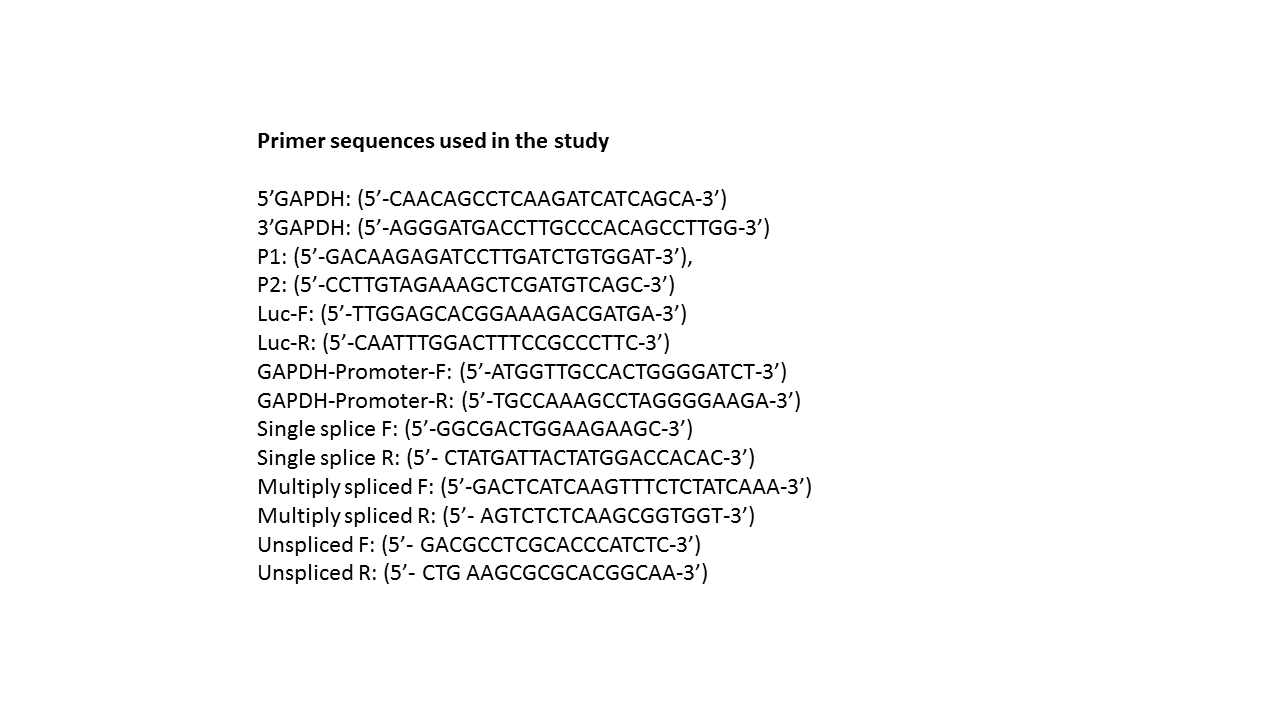

Supplement: TEXT S1 [file mbo005184147s1.tif]
